# The Ageing Brain: Molecular and Cellular Basis of Neurodegeneration

**DOI:** 10.3389/fcell.2021.683459

**Published:** 2021-08-13

**Authors:** Shofiul Azam, Md. Ezazul Haque, Rengasamy Balakrishnan, In-Su Kim, Dong-Kug Choi

**Affiliations:** ^1^Department of Applied Life Sciences, Graduate School, BK21 Program, Konkuk University, Chungju-si, South Korea; ^2^Department of Biotechnology, College of Biomedical and Health Science, Research Institute of Inflammatory Disease (RID), Konkuk University, Chungju-si, South Korea

**Keywords:** neurodegenerative diseases, NAD^+^, aggregation, mitophagy, inflammation

## Abstract

Ageing is an inevitable event in the lifecycle of all organisms, characterized by progressive physiological deterioration and increased vulnerability to death. Ageing has also been described as the primary risk factor of most neurodegenerative diseases, including Alzheimer’s disease (AD), Parkinson’s disease (PD), Huntington’s disease (HD), and frontotemporal lobar dementia (FTD). These neurodegenerative diseases occur more prevalently in the aged populations. Few effective treatments have been identified to treat these epidemic neurological crises. Neurodegenerative diseases are associated with enormous socioeconomic and personal costs. Here, the pathogenesis of AD, PD, and other neurodegenerative diseases has been presented, including a summary of their known associations with the biological hallmarks of ageing: genomic instability, telomere attrition, epigenetic alterations, loss of proteostasis, mitochondrial dysfunction, cellular senescence, deregulated nutrient sensing, stem cell exhaustion, and altered intercellular communications. Understanding the central biological mechanisms that underlie ageing is important for identifying novel therapeutic targets for neurodegenerative diseases. Potential therapeutic strategies, including the use of NAD^+^ precursors, mitophagy inducers, and inhibitors of cellular senescence, has also been discussed.

## Introduction

Ageing is an inevitable event of the lifecycle of an organism, associated with physical deterioration and an increased risk of disease and death (Hou et al., 2019). The deterioration rate in ageing differs across species, individuals, and tissues (Carmona and Michan, 2016). As the ageing populations increases, the financial burden associated with age-related health issues also increases; therefore, slowing or preventing the development of age-related health problems represents an urgent need. Among the various diseases encountered during ageing, neurodegenerative diseases and their associated cognitive deficits are prevalent among older populations, affecting their healthy lifespan and quality of life. Neurodegeneration is a complicated brain disorder that is not yet fully understood. Among the many of risk factors that have been associated with neurodegeneration, ageing biomarkers are by far the most relevant (Wyss-Coray, 2016). Nine critical hallmarks of the ageing process have been identified in recent years (López-Otín et al., 2013; Hou et al., 2019), each of which has been associated with the pathogenesis of at least one neurodegenerative disease (Hou et al., 2019), which will be discussed critically here. The prevalence of neurodegenerative diseases among older populations is so common that disease-free brains are rare. Suggesting, brain ageing might be a scale of neurodegeneration progression, and human genetic and environmental factors may serve as determinants for the onset and progression of neurodegenerative disease (Wyss-Coray, 2016).

Hallmarks of ageing, including mitophagy, cellular senescence, genomic instability, and protein aggregation, have been associated with various neurodegenerative diseases, including Alzheimer’s (AD) and Parkinson’s disease (PD) (Hou et al., 2019). Whether ageing-related changes in the brain are harbingers of neurodegeneration is not clear. Additionally, the most prevalent neurodegenerative diseases, AD and PD, share the common feature of protein aggregation, despite their various other clinical symptoms. The aggregation of senile plaques containing extracellular amyloid-beta (Aβ) peptide and the formation of intraneuronal tau containing neurofibrillary tangles (NFTs) in AD and the accumulation of misfolded α-synuclein (α-syn) in PD are major pathogenic features of these diseases (Bourdenx et al., 2017). Protein aggregation is also a feature of amyotrophic lateral sclerosis (ALS) and frontotemporal lobar dementia (FTD); the 43-kDa TAR DNA-binding protein (TDP-43) aggregates in ALS and the microtubule-associated protein tau aggregates in FTD and AD (Ransohoff, 2016). This study aimed to synthesize the current knowledge regarding ageing-related research and to correlate these findings with neurodegenerative disorders to highlight prospective and novel therapeutic avenues.

## Etiologies of Brain Ageing and Neurodegeneration

**FIGURE 1:**
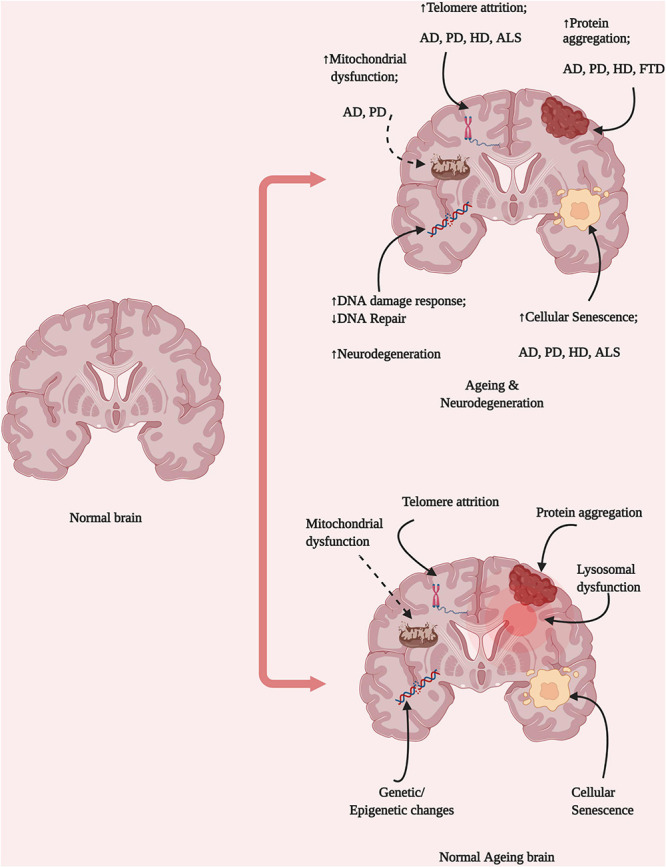
Brain ageing hallmarks and neurological diseases. Ageing represents the combined disruption of several homeostatic processes, including protein aggregation, DNA damage, mitochondrial dysfunction, lysosomal dysfunction, and changes in epigenetic regulation. These changes might be dependent on different cell types and result in the development of various diseases depending on their origins in different brain locations and the pattern of propagation (Created with BioRender.com).

Brain ageing is an irreversible process, and disease-free brains among older populations are rare, one in 10 ≥ 65 years old people have AD (Hou et al., 2019). Neurodegeneration is among the most prevalent of age-related diseases, which suggests the existence of a link between neurodegenerative diseases and ageing-related changes that occur in the brain microenvironment, such as genomic instability, epigenetic modifications, and the loss of proteostasis (Figure 1). Although ageing is known to be a major risk factor for neurodegenerative diseases, the exact mechanisms through which ageing is associated with neurodegeneration have not yet been identified. Molecular studies have identified that proteins like α-synuclein, phosphorylated tau and Aβ aggregates abnormally with ageing, however, it is not confirmed that they are associated with cognitive impairment or not (Elobeid et al., 2016). Some studies indicated that early life developmental defects of brain are associated with neurodegenerative disease development risk and in that case cognitive impairment might take place lately (Dean et al., 2014). Exposure to environmental stimulus like trauma, drugs or toxins have harsh consequences in later life including defective neuroplasticity and cognitive impairment (Schaefers and Teuchert-Noodt, 2016). Present review, therefore, attempted to correlate biological factors of normal brain ageing and risk to develop neurodegenerative diseases and discusses therapeutic avenues.

Cellular longevity has consistently been linked to the *Tomm40*–*Apoe*–*Apoc1* locus, *Foxo3*, and *IL6* (Erikson et al., 2016; Matteini et al., 2016; Wyss-Coray, 2016; Zeng et al., 2016). A large meta-analysis of many of single-nucleotide polymorphisms (SNPs) linked to longevity with the greatest significance were negatively linked to AD (Sebastiani et al., 2013). Interestingly, healthy ageing, which refers to ageing without disease development may not be associated with longevity genes but instead may be associated with the absence of AD risk factors (Erikson et al., 2016). A cohort study of the genetic sequences obtained from individuals aged over 80 years without the development of chronic diseases revealed that many SNPs are linked to the regulation of cognitive performance, suggesting that brain health and cognition are related and genetic factors may determine whether an individual undergoes healthy ageing or develops a neurodegenerative disease (Sebastiani et al., 2013).

Brain tissues are composed primarily of postmitotic cells, including neurons and oligodendrocytes, which are highly sensitive to age-dependent changes, such as DNA damage or methylation. DNA methylation is an epigenetic mark that involves DNA methyltraferase-mediated transfer of methyl group to the C-5 position of the cytosine ring of DNA (Jin et al., 2011; Horvath, 2013). In mammalian, ≥98% DNA methylation takes place in CpG dinucleotide in somatic cells, whereas approximately a quarter of total methylation occurs in non-CpG in embryonic cells (Lister et al., 2009). Ageing has been shown to alter the DNA methylation process, resulting in DNA damage, which might contribute to the etiology of neurodegeneration. For example, the blood-based analysis (intrinsic and extrinsic epigenetic age accelerated blood count; as DNA methylation based biomarker) of PD patients showed that consistent DNA methylation patterns were associated with advanced ageing (Horvath and Ritz, 2015); epigenetic age of immune system significantly increased in PD, where granulocyte is playing a major role. Two independent studies identified that DNA methylation at sites close to *Ank1*, *Cdh23*, *Rhbdf2*, and *Rpl13* may be linked to AD pathology (De Jager et al., 2014; Lord and Cruchaga, 2014; Lunnon et al., 2014); except for *Cdh23*, these genes are strongly linked to the AD-associated gene *Ptk2b*. Advanced ageing is also associated with increased mitochondrial dysfunction and damage, which promotes neurodegeneration via the generation of reactive oxygen species (ROS) and the promotion of neuroinflammation.

## Hallmarks of Brain Ageing

The nine-biological hallmarks of ageing have been broadly categorized into primary, antagonistic, and integrative. Primary hallmarks of ageing include genomic instability, epigenetic alterations, telomeric attrition, and the loss of proteostasis. Antagonistic hallmarks refer to compensatory responses to primary damage associated with ageing, including mitochondrial dysfunction, cellular senescence, and the downregulation of nutrient sensing. Integrative hallmarks are the results of cumulative damage of primary and antagonistic hallmarks and include stem cell exhaustion and altered intercellular communications.

### Primary Hallmarks

Approximately 10^5^ DNA lesions in the human genome are generated in each cell daily (Lindahl, 1993) due to exposure to endogenous ROS or reactive nitrogen species (RNS) or environmental hazards, such as radiation, chemical mutagens, or carcinogens. DNA damage can include bulky adducts, abasic sites, single-strand breaks, double-strand breaks, insertions, and deletions, which have all been associated with neurodegenerative diseases (Jeppesen et al., 2011). The key endogenous repair mechanisms are nucleotide excision repair (NER), base excision repair (BER), mismatch repair, double-strand break repair, and direct reversal (Jeppesen et al., 2011). However, when the demands for DNA repair exceed the normal capacity, replication fork blockages can develop resulting in the translation of error-prone DNA polymerases (Zeman and Cimprich, 2014), which is a major pathogenic contributor to premature ageing and neurodegeneration in humans (Lombard et al., 2005). Mutations to genes involved in the BER pathway can cause ageing to accelerate and increase the risk of neurodegeneration (Leandro et al., 2015). Exponential DNA damage causes genomic instability and initiates a signaling cascade that permeates throughout the cell. Poly (ADP-ribose) polymerase (PARP1) is an abundant protein that activates the PARylation process, in which PAR polymers accumulate at DNA damage sites, and cleaves large quantities of NAD^+^ (Bai, 2015; Bai et al., 2015). Continuous DNA damage requires large amounts of energy to accelerate the repair process, and NAD^+^ is an essential cofactor necessary for the maintenance of DNA repair, mitophagy, and mitochondrial health. DNA damage can exacerbate cellular senescence and inflammation, triggering ageing-related neurodegeneration.

A defective telomere is another primary hallmark of ageing, which has been identified in mice and humans (López-Otín et al., 2013). Telomeres are chromosomal end regions, composed of protein and DNA and may impact the pathogenesis of neurodegeneration (Fang et al., 2017). Telomeres become shorter over time unless parent cells express telomerase to prevent telomere attrition, and telomere shortening has been shown to accelerate ageing. Short telomeres induce cellular senescence and have been associated with diseases, such as osteoarthritis, atherosclerosis, and atrial fibrillation (Hunt et al., 2015; Kuszel et al., 2015).

Epigenetic modifications, including DNA methylation, PARylation, and acetylation, represents an emerging interest in the field of ageing and neurodegeneration (Hwang et al., 2017). Epigenetic modifications can strongly affect chromatin functions, including transcription and replication.

Proteostasis refers to the mechanisms involved in the maintenance of proteome homeostasis and the regulation of protein turnover (Eisenstein, 2014). In mammals, proteostasis mechanisms serve to prevent protein misfolding and aggregation. In eukaryotic cells, several processes contribute to proteostasis, including autophagy, ubiquitination, and the lysosomal system. Autophagy is a degradation process that facilitates the lysosomal process and reduces the levels of inflammatory cytokines triggered by misfolded proteins or damaged organelles (Tanaka and Matsuda, 2014). Frequent aggregation of misfolded protein is common in different neurodegenerative diseases like AD and PD (Espay et al., 2019).

### Antagonistic Hallmarks

Neuronal cells require high energy levels to perform neuronal functions and are highly affected by mitochondrial changes. The healthy mitochondrial pool is maintained by the fission and fusion of individual mitochondria and the removal of damaged mitochondria through mitophagy (Fang et al., 2016). Rapid DNA damage increases the cellular energy requirements, stimulating mitochondrial fusion and depleting NAD^+^. The impaired restoration of NAD^+^ levels can negatively affect the cellular redox balance, exacerbating oxidative stress (Blacker and Duchen, 2016) and resulting in stress-induced neurodegeneration. Brain mitochondrial functions change with ageing and are thought to be major contributors to the ageing process and most known neurodegenerative diseases.

Mitophagy is a natural process for the removal of defective or damaged mitochondria. One known mitophagy pathway is the PTEN-induced putative kinase protein 1 (PINK1)–Parkin pathway, in which PINK1 accumulates on the outer membrane of a depolarized mitochondrion and recruits the E3 ubiquitin ligase Parkin. Parkin ubiquitinates mitochondrial proteins, targeting the damaged mitochondrion for degradation. Damaged organelles and proteins can be degraded and cleared from cells by the autolysosome, which is formed by the fusion of the autophagosome with the lysosome. Both PINK1 and Parkin play important roles the regulation of mitochondrial function and the elimination of intracellular Aβ aggregates from the AD brain (Khandelwal et al., 2011; Du et al., 2017), and mutations in the PINK1- or Parkin-encoding genes are associated with locomotor deficits in early onset PD and can cause locomotor deficits in *Drosophila melanogaster* and humans (Pickrell and Youle, 2015). A decrease in mitophagy-related proteins, like phosphorylated-TANK binding kinase 1 (p-TBK1) and p-ULK1, promotes the accumulation of defective mitochondria and impairs cellular energy production. However, mitophagy can occur independently from PINK1 and Parkin, and at least 20 mitophagy-regulating proteins in mammals have been reported, thus far (Kerr et al., 2017), including the serine/threonine-protein kinase ULK1, BCL2/adenovirus E1B 19kda protein-interacting protein 3-like (BNIP3L/NIX), the serine/threonine-protein kinase TBK1, and FUN14 domain-containing 1 (FUNDC1). A recent study demonstrated that AD associated protein Aβ_1–4__2_ and p-tau are major contributors to mitophagy dysfunction (Fang et al., 2019). Mitophagy dysfunction also impairs ATP production, which can promote tau phosphorylation by activating 5′ AMP-activated protein kinase (AMPK) and excessive mitochondrial fission (Fang et al., 2019). These findings suggest that mitophagy is important for the mitigation of inflammation, which is a major factor in neurodegenerative progression. A study showed that the overexpression of Nip3-like protein X (NIX) could improve mitochondrial ATP production in cells derived from PD patients, even in the absence of PINK1 and Parkin (Koentjoro et al., 2017). These findings suggested that even in cases of PINK1/Parkin-encoding gene mutation, mitophagy could be restored, indicating that mitophagy-stimulating agents may represent a potential therapeutic strategy for several neurological diseases.

Cellular senescence refers to a stress-induced state of stable cell cycle arrest, and a senescence-associated secretory phenotype (SASP) occurs with age (Hayflick, 1965). SASP is a survival process for healthy cells, facilitating the removal of damaged cells under stressful conditions (Kültz, 2005). Cellular senescence is typically a tumor-suppressing process; however, during advanced ageing, senescence could serve as a tumor initiator (Loaiza and Demaria, 2016). Cells undergoing mitochondrial-associated senescence have been characterized with a decreased NAD^+^:NADH ratio, halting growth and reducing IL-1-mediated SASP induction (Wiley et al., 2016). NAD^+^ metabolism is an important regulator of SASP via the high mobility group A (HMGA)-nicotinamide phosphoribosyltransferase (NAMPT)-NAD^+^ signaling pathway, which regulates proinflammatory SASP (Nacarelli et al., 2019). Cells that feature a high level of damaged DNA become senescent, with reduced proliferation. Because ageing is associated with a reduced DNA repair capacity and increased DNA damage, damaged DNA accumulate, increasing energy demands and ROS levels in neuronal cells (Madabhushi et al., 2014; Maynard et al., 2015). Neuronal cells with consistently active DNA damage repair mechanisms demonstrate many cellular senescence features, and these neurons are called senescence-like neurons (Fielder et al., 2017). This senescence-like state has been observed in a mouse model of ageing (C57BL/6), in which 40–80% of Purkinje neurons and 20–40% of cortical, hippocampal, and peripheral neurons were characterized by high levels of DNA damage, oxidative stress, and senescence-associated β-galactosidase activity (Jurk et al., 2012). Researchers have suggested that advanced ageing not only reduces the DNA repair capability but also introduces complex DNA repair mechanisms that might promote additional mutations (Vaidya et al., 2014). Autophagy is another important process that has been associated with cellular senescence (Narita et al., 2011); however, the role played by autophagy remains under debate. For example, macro-autophagy might contribute to the establishment of SASP via the synthesis of secretory proteins (Gewirtz, 2013), whereas autophagy inhibition may promote senescence via ROS and p53, under some circumstances (Kang et al., 2011). Because the cellular senescence process accelerates ageing and promotes ageing-associated brain disorders, senescence could be a potential therapeutic target for neurodegenerative diseases.

Caloric restriction has been shown to have lifespan-extending effects in mice by downregulating nutrient signaling pathways, and caloric restriction has been suggested to be neuroprotective in humans (Fontana et al., 2010). Major nutrient-sensing pathway components include insulin, insulin-like growth factor 1 (IGF1), mechanistic target of rapamycin (mTOR), AMPK, and sirtuins (López-Otín et al., 2013; Hou et al., 2019). Dysfunction in one or several metabolic pathways is a common feature of neurodegenerative disorder patients, which may result in low NAD^+^ levels and mitochondrial dysfunction (Babbar and Sheikh, 2013; Hou et al., 2019).

### Integrative Hallmarks

Functional stem cells are essential for a healthy lifespan. According to recent research, functional and proliferative stem cells decline during advanced ageing. Ageing limits stem cell division and renewal, which be associated with the lack of DNA repair ability, frequent DNA damage, or environmental cues, including stressful conditions, diseases, proteostasis, epigenetic changes, cellular senescence, and mitochondrial dysfunction (Mantovani et al., 2014; Oh et al., 2014). Mitochondria are active regulators of stem cell pluripotency, controlling cell fate by supporting various metabolic pathways and ROS production. ROS are generated by electrons produced by mitochondrial oxidative phosphorylation that leak into the cytoplasm. ROS are major driving factors of stem cell exhaustion (Oh et al., 2014); the decline in stem cells number and function, which is maintained by adult stem cells of all tissues and organs including forebrain and muscles (Ren et al., 2017). The accumulation of cellular damage and mitochondrial dysfunction promote ROS production, which, in turn, exacerbates cellular macromolecular damage and dysregulates mitochondrial oxidative phosphorylation, causing cellular decomposition (Harman, 1972; Oh et al., 2014). Villeda et al. (2014) demonstrated that ageing downregulates synaptic plasticity-related gene expression, resulting in reduced spine densities and impairments in cognitive function. Researchers have identified a connection between synaptic plasticity-related transcriptional changes that occur during ageing and the circulatory system, and the infusion of young blood plasma, derived from young mice, can improve dendritic spine densities in mature neurons and enhance synaptic plasticity in the hippocampus of aged heterochronic parabionts (Villeda et al., 2014), where young and aged animals circulatory systems are connected. Plasma concentrations of glycosylphosphatidylinositol (GPI)-specific phospholipase D1 (*Gpld1*), a liver-derived enzyme, increase after exercise and is abundant in the plasma of active, healthy, older individuals, and *Gpld1* has been associated with improvements in cognitive function (Horowitz et al., 2020). A study suggested that the reversal of brain ageing might be possible by transferring post-exercise blood to older adults (Horowitz et al., 2020). That same study suggested that the blood cofactor *Gpld1* transfers from the liver to the brain, where it regulates cognitive functions. Blood transfusion through heterochronic parabiosis resulted in improved stem cell function in the brain, which countered ageing-related deficits and rejuvenated cognitive functions; therefore, aged stem cells could serve as a potential therapeutic target for alleviating ageing-related neurodegeneration.

Understanding the body’s intrinsic mechanisms for detecting neurodegeneration and protecting the brain from progressive neurodegenerative damage is important for understanding how these processes become disrupted. Microglia, which are the resident immune cells of the brain, play important roles in both the progression and prevention of neurodegenerative disease development. During ageing, prominent increases in chemokine-enriched microglia populations may be triggered by compromised blood–brain barrier function or the occurrence of microinfarcts (Hammond et al., 2019). Disease-associated microglia (DAM) can sense early phases of CNS damage through various signaling molecules, including *Trem2* (Deczkowska et al., 2018). Ageing also alters other hormones, including leptin, ghrelin, insulin, and adiponectin, that regulate neuronal damage and neurodegeneration. However, pathological evidence from neurodegenerative diseases has suggested that microglia interact with other glial supportive cells, including astrocytes and oligodendrocytes (Amor and Woodroofe, 2014). Cross-talk between mast cells, T cells, microglia, astrocytes, and oligodendrocytes is an important contributor to ageing-related functional changes in the immune system and neurodegenerative diseases.

Inflammation refers to the protective and defensive response of an organism against a variety of exogenous insults and can become upregulated and uncontrolled during advanced ageing. Inflammatory responses can be beneficial as acute, transient reactions to harmful conditions, promoting the defense, repair, and adaptation of host tissues. However, chronic and low-grade inflammation is detrimental for many tissues and likely to hamper normal functions (Calder et al., 2017). Along with persistent microglial activation, sustained increases in proinflammatory cytokines, and elevated levels of oxidative stress, chronic inflammation has become a hallmark of ageing-related neurodegeneration (Calder et al., 2017; Hickman et al., 2018). Chronic microglial activation increases the production of proinflammatory cytokines, such as IL6, IL-1β, and TNF-α. ROS generated by dysfunctional mitochondria also activates nuclear factor kappa B (NF-κB)-mediated inflammatory signaling pathways during ageing and potentiate the NOD-, LRR- and pyrin domain-containing protein 3 (NLRP3) inflammasome (Haque et al., 2020). Inflammation also exacerbates Aβ deposition in AD and α-syn truncation and aggregation in PD (Wang et al., 2015; Wang W. et al., 2016). Therefore, further exploration of the disease-specific protein misfolding that occurs in response to inflammation during ageing-related disease progression could identify potential therapeutic targets.

Other factors associated with inflammation include defective proteasome and autophagy degradation systems (Grune et al., 2004; Nakahira et al., 2011), cellular senescence (Baker and Petersen, 2018), increased oxidative DNA damage, impaired DNA repair (Pan et al., 2016), and decreased innate and adaptive immune responses (Frasca and Blomberg, 2016). Targeting these pathway(s) could regulate ageing and ageing-associated neurodegeneration.

## Ageing and Neurodegenerative Diseases

### Alzheimer’s Disease

**FIGURE 2:**
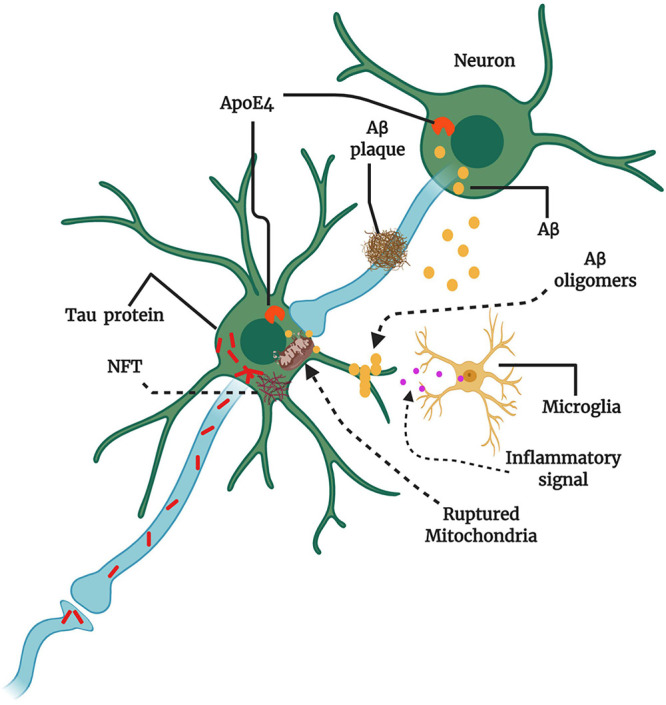
Key players and therapeutic targets in AD pathogenesis. The production of amyloid-beta (Aβ) instead of clearance can cause Aβ oligomerization. Aβ oligomers transmit through synapses, and Aβ plaque formation may hinder synaptic plasticity and transmission. The abundant intracellular Aβ can surround mitochondria and impair mitochondrial function, resulting in the release of reactive oxygen species (ROS), the activation of microglia, and the production of inflammatory signals. Microglial activation can be both beneficial and detrimental depending on the engaged signaling cascade. The protein tau also may aggregate and form large intraneuronal pathogenic aggregates known as neurofibrillary tangles (NFTs). Therapies might target amyloid precursor protein (APP) in the neuronal membrane to reduce Aβ production, increase Aβ clearance from synapses, and reduce Aβ oligomer formation. Apolipoprotein E4 (ApoE4) and tau can also promote Aβ production. Misfolded tau within neurons aggregate into NFTs, and misfolded tau can be transmitted through the synapse to affect other neurons. Therapies could be designed to reduce tau aggregation or misfolding. Multiple factors are involved at various stages of AD progression, damaging neuronal circuits and causing neuronal loss and neurological decline (Conceptualized from 58, Created with BioRender.com).

Neurological diseases, such as AD, has long been easy to diagnose but difficult to treat because the molecular pathogenesis was unknown. The advanced research techniques and new methods have revealed various molecular and cellular pathways that are thought to be involved in AD pathogenesis (Figure 2), although no cure has yet been identified. Several ageing hallmarks, such as genetic mutations, epigenetic modifications, cellular senescence, and altered intercellular communications, have also been implicated in AD progression. Faulty DNA repair machinery and increased DNA mutation are key factors in AD. Post-mortem brain analyses have revealed the loss of gene expression, such as *Pol*β, β*-OGG1* glycosylase, and uracil DNA glycosylase (Weissman et al., 2007). The downregulation of *Pol*β has been associated with neuronal loss, synaptic dysfunction, and cognitive impairments (Sykora et al., 2015).

At least three proteins defect has been suggested as core risk factor for AD (Bekris et al., 2010), which are amyloid precursor protein (APP), presenilin 1 (PSEN1), and presenilin 2 (PSEN2). Approximately 1–5% of all AD cases can be linked to autosomal dominant genetic mutations of these protein encoding genes, which is termed as familial or early onset AD. Several highly penetrant mutations have been identified in genes at a variety of chromosomal locations, including *APP* (chromosome 21q21.2), *PSEN1* (chromosome 14q24.3), and its homolog *PSEN2* (chromosome 1q42.13) (Goate et al., 1991; Rogaev et al., 1995; Sherrington et al., 1995). In addition, the complex interaction between genetic and environmental factors has been suggested as the core risk factor for sporadic or late-onset AD. Allelic variants of apolipoprotein E (*ApoE*) have also been associated with the increased susceptibility of sporadic AD (Roberson and Mucke, 2006; Figure 2). According to risk assessments, ApoE-ε4 homozygotes showed a greater than 50% risk of AD development, whereas ApoE-ε3 and ApoE-ε4 heterozygotes were associated with a 20–30% risk of developing AD in 11% of men and 14% of women overall, irrespective of ApoE allele combination (Philip et al., 2016). Genome-wide association studies (GWAS) and next-generation sequencing (NGS) have uncovered interesting *Trem2* [p.R47H (rs75932628) and p.R62H (rs143332484)] and *Plcg2* [p.P522R (rs72824905)] variants associated with sporadic AD risk factors (Sims et al., 2017; van der Lee et al., 2019). Another study reported 56 genes involved in the immune system that were associated with the development and progression of AD, including microglial-expressed *Tyrobp* and *Trem2* (Sims et al., 2017). The Trem2/Tyrobp signaling cascade modulates tau phosphorylation and Aβ production in transgenic mice that express P301S mutant tau (Paris et al., 2014).

The major molecular features of AD are aggregated Aβ plaques and p- tau neurofibrillary tangles (NFTs) in the brain (Bloom, 2014). The sequential cleavage of APP by β- secretase and γ- secretase enzymes at N-terminus and C-terminus (respectively) produces shorter and abnormal Aβ fragments (36–43 amino acids of APP) (Wang J. et al., 2017). Although the primary pathological factors of AD are still under debate, but it seems to include cholinergic dysfunction, Aβ plaques, tau aggregation, inflammation, DNA damage and mitochondrial dysfunction. Thereby, current therapeutics approved by FDA (so far) are mostly acetylcholinesterase inhibitors such as donepezil, galantamine and rivastigmine (Lane et al., 2018). But these drugs alleviate some symptoms only and showed no or limited disease prevention ability.

Hardy and Higgins (1992) proposed that Aβ deposition (oligomers and fibrils accumulation) is the first step of AD pathogenesis, leading to subsequent tau deposition, neuron and synaptic loss, and cognitive deficits. Although some portion of this Aβ hypothesis has been modified with time course, but still it is a leading model for AD therapeutics development. Based on this hypothesis, many potential anti- Aβ drugs has been developed and are under development, unfortunately many of them have failed in clinical trials. For example, verubecestat (BACE inhibitor) showed lack of efficacy and toxicity in preclinical AD participants, CNP520 (BACE inhibitor) worsens cognition in early AD participants (Huang et al., 2020). Phosphorylated- tau protein aggregation in neurons causes neuronal damage and leads to AD (Hou et al., 2019), thereby, it has been considered as another potential drug targets of AD. Some agents like TRx0237 (anti-tau aggregation) and ANAVEX2-73 (anti-tau and anti-Aβ) has been developed and under clinical trial (Huang et al., 2020). The Aβ and/or p- tau aggregation, oxidative stress, and internal or external stress can manipulate brain immune response and results in neuroinflammation, which is to considered as another key driver of AD (Ransohoff, 2016). Neuroinflammatory responses are generally initiates from abnormal activation of astrocytes and microglia that releases pro- inflammatory cytokines. The other risk factors of AD are mitochondrial dysfunction and DNA damage. As average brain weighs 2% of total body weight but uses 25% of total body glucose, impaired energy metabolism due to mitochondrial dysfunction seems to fuel clinical onset of AD. Mitochondrial dysfunction results in disruption of mitochondrial biogenetics, mitochondrial genomic homeostasis and increases oxidative stress that are linked to AD pathogenesis (Wang et al., 2020). Also, impaired mitochondrial fission and fusion balance have an essential role in mitochondrial dysfunction and AD pathogenesis (Wang et al., 2014; Wang W. et al., 2017). As of now, many of potential drugs developed on the basis of Aβ hypothesis has been failed in clinical trial, which is suggesting looking into other risk factor/s to intervene AD progression.

Epigenetic modifications, such as DNA methylation, PARylation, ubiquitination, and acetylation, play important regulatory roles in AD progression and the associated learning, behavior, and memory deficits (Wang H.-Z. et al., 2016). For example, the phosphorylation or hyperphosphorylation of histone H3 and the deacetylation of histone H4 have been detected in the hippocampus of the early stage AD brain (Esposito and Sherr, 2019).

Accumulation of damaged mitochondria due to impaired mitophagy is one of major hallmark of ageing and ageing-related neurodegenerations like AD, PD and others (Fang et al., 2019). A recent study showed that stimulation of mitophagy (via NAD^+^ supplementation and actinonin) mitigates neuroinflammation and abolishes insoluble Aβ fragments (1–40 and 1–42) in APP/PS1 mouse model, also diminished hyperphosphorylation of tau in human iPSC-derived human neurons (Fang et al., 2019). Telomere instability or shortening could be a risk factor for AD development as well. Both ageing mice and human has shown defective telomere maintenance, and similar feature also been evident in AD patients (Herrmann et al., 2018). A meta-analysis of 13 studies demonstrated a significant difference in telomere length between 860 AD patients and 2,022 controls (Forero et al., 2016), suggesting a consistent shortening of telomere in AD. The final evidence indicating to highlight analysis of epigenomic markers associated with ageing-related neurodegeneration, such as AD.

During advanced ageing, stem/progenitor cell dysfunction and cellular senescence often develop, associated with chronic ageing-related disease pathogenesis. Oligomerized Aβ_1–4__2_ has been demonstrated to trigger neural stem/progenitor cells (NSPCs) senescence (He et al., 2013). Aβ_1–4__2_ oligomerization potentiates the expression of the senescence-associated markers p16 and senescence-associated β-galactosidase (SA-β-gal) in the hippocampus. Formylpeptide receptor 2 (FPR2) and ROS-responsive p38 mitogen-activated protein kinase (MAPK) have also been implicated in this phenotypic senescence modification in the hippocampus (He et al., 2013), which may be associated with neurogenesis failure and related memory decline in AD.

### Parkinson’s Disease

PD is a progressive, neurodegenerative movement disorder that is linked to advanced ageing. The incidence of PD increases exponentially from age 45 to 100 years. A cohort study showed that the age-specific incidence of PD peaks at 85 years or older, whereas the risk of PD development begins to increase starting at approximately 75 years of age (Driver et al., 2009). The molecular pathogenesis of PD includes neuronal loss from the substantia nigra region due to α-syn proteostasis, oxidative stress, mitochondrial dysfunction, disrupted axonal transport, and neuroinflammation (Poewe et al., 2017). Together, these features can cause striatal dopamine deficits, which manifest as locomotor symptoms, including impaired movement amplitudes and speeds, limb rigidity, or resting tremor.

**FIGURE 3:**
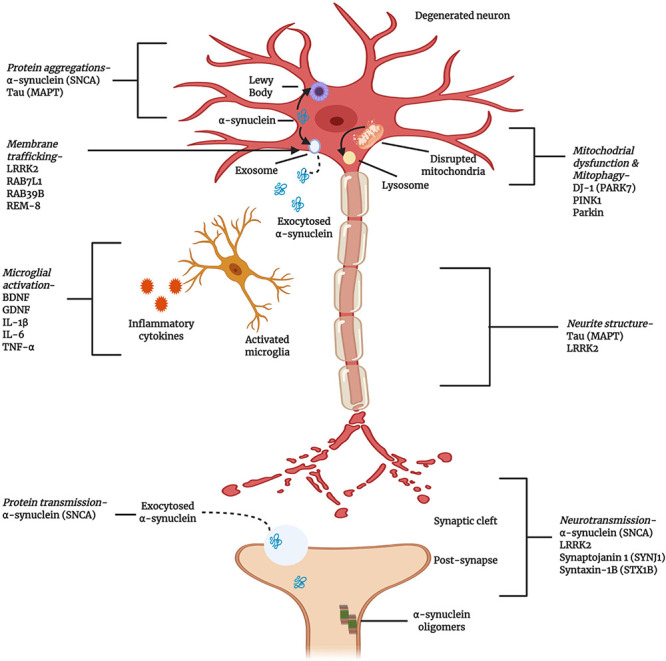
Cellular and molecular activities associated with the pathogenesis of PD. Multiple genes have been implicated, either as direct genetic causes of familial Parkinson’s Disease (PD) or as polymorphisms that have been identified as risk factors for the development of sporadic PD. The gene products highlighted here are key to the progress and pathogenesis of PD, although this figure is not exhaustive (Created with BioRender.com).

The first PD-associated genetic discovery occurred in 1997 when a missense variant in *SNCA* (α-syn) was identified (Polymeropoulos et al., 1997). Since then, family-based studies have continued to provide a productive line of investigation. To date, missense and loss of function mutations in approximately 20 genes have been correlated with PD, including *PRKN* (missense or loss of function), *PARK7* (missense), *LRRK2* (missense), *PINK1* (missense or loss of function), *PLOG* (missense or loss of function), and *GBA* (missense or loss of function) (Blauwendraat et al., 2020). Therefore, recent pharmacological developments have focused on the restoration of striatal dopamine levels through gene- and cell-based approaches, and α-syn aggregation and cellular transportation have been identified as the therapeutic targets thought to have the most potential (Poewe et al., 2017; Figure 3).

Many studies have demonstrated that the aggregations of α-syn is neurotoxic and can spread throughout the brain, exaggerating the disease pathology (Brettschneider et al., 2015). All PD patients exhibit neuronal α-syn aggregation. During early stage PD, soluble α-syn exists in monomeric form. The formation of α-syn into oligomers eventually leads to the formation of large, insoluble, neurotoxic fibrils known as Lewy bodies (Melki, 2015). These α-syn aggregates and be transmitted cell-to-cell during PD progression. Mutations in the 140-amino-acid α-syn protein play distinct roles in this transmission process. PD patients with α-syn mutations associated with the early onset disease have more severe pathological symptoms than sporadic PD patients expressing wild-type α-syn. A study demonstrated that specific mutations that affect the α-syn protein structure, such as A53T, H50Q, and G51D, can promote the propagation of synucleinopathy and induce neuroinflammation in the substantia nigra pars compacta region of rats (Guan et al., 2020).

Mitochondrial dysfunction is another feature of PD pathogenesis, associated with α-syn aggregation and transmission. In addition to mutations in *SNCA*, other genes, such as *PINK1*, *Parkin*, and *LRRK2*, have been directly and indirectly associated with both familial and sporadic PD. These genes modulate mitochondrial function and morphological integrity (Li et al., 2020; Figure 3). PINK1/Parkin signaling plays an important role in mitochondrial quality control, including through the activation of mitophagy, autophagy-based clearance mechanism for damaged mitochondria. In general, PINK1 is located at the inner mitochondrial membrane but translocates to the outer membrane after sensing severe mitochondrial degradation, where it phosphorylates and activates the outer membrane protein Parkin. Parkin ubiquitinates mitochondrial proteins, targeting mitochondria for mitophagy. The PINK1/Parkin signaling pathway is highly conserved in both humans and *Drosophila*, and mutations in either gene cause damage to dopaminergic neurons (Shiba-Fukushima et al., 2017). This signaling pathway also regulates the mitochondrial fission/fusion ratio, and mutations in either *PINK1* or *Parkin* results in altered phenotypes, such as muscle degradation, motor deficits, and mitochondrial morphological alterations. For example, in *Drosophila* and rats, the overexpression *PINK1* was associated with excitotoxicity that could be suppressed by the overexpression of dynamin-related protein 1 (*Drp1*) (Yu et al., 2011). Though the exact beneficial and detrimental roles associated with this signaling pathway remain unclear, *PINK1/Parkin* signaling plays important roles in neuronal mitochondrial dynamics and function.

The hallmark of PD pathology is the formation of Lewy body inclusions, composed of α-syn. PD patients also showed reduced mitochondrial complex I expression. Interestingly, the deletion of α-syn protein in mice also reduced mitochondrial complex formation; thus, the α-syn protein balance in mitochondria is important for normal mitochondrial function (Devi et al., 2008). Both wild-type and mutant α-syn aggregation can cause mitochondrial protein modifications, eventually resulting in the loss of dopaminergic neurons. In *Drosophila*, α-syn accumulation has been reported to alter the actin cytoskeleton and *Drp1* localization, resulting in mitochondrial dysfunction (Ordonez et al., 2018). The PD protein 7 (PARK7), also known as DJ-1, serves as a transcriptional regulator, antioxidant scavenger, and redox sensor in the mitochondria. DJ-1 can clear remove mitochondrial α-syn protein aggregation, and *DJ-1* mutations are associated with early onset familial PD (Bonifati et al., 2003).

Cellular senescence has been associated with sporadic PD development and α-syn deposition in PD brains. An increased number of senescent cells in PD patients has been associated with increased SA-β-gal and p16 activity, resulting in increased proinflammatory cytokine IL6 secretion (Chinta et al., 2018).

Adult neurogenesis in the hippocampus, subventricular zone, and other regions is reduced during early stage PD (Regensburger et al., 2014). Inducible pluripotent stem cells (iPSCs) are a promising source for cell-based therapies, and the transplantation of human iPSCs into primate dopaminergic neurons resulted in the development of dense neurites in the striatum, similar to healthy individuals (Kikuchi et al., 2017).

Neuroinflammation is another emerging field that has been the focus of pharmacological interventions for neurodegenerative diseases. Autopsy examinations performed on tissue samples from PD patients have revealed distinct α-syn aggregates in glial cells which have been shown to be associated with the generation of proinflammatory mediators (Ransohoff, 2016). In addition to glial cell activation, fibrillary α-syn increases IL-1β secretion through TLR2 interaction, which is involved in NLRP3 inflammasome activation (Codolo et al., 2013).

### Multiple Sclerosis

In young adults, multiple sclerosis (MS) is the most frequently diagnosed neuroautoimmune disorder, associated with severe, physical, non-traumatic disability. Well-defined neuroinflammatory demyelinating lesions and neuronal loss are the characteristic hallmarks of MS. MS onset occurs when natural killer cells, T and B lymphocytes, and dendritic cells infiltrate the spinal cord and brain through a disruption in the blood–brain barrier (BBB), which worsens over time (Frischer et al., 2009).

Although the exact role of DNA methylation in MS is yet not fully understood, but several studies have shown differentially methylated regions in immune cells and brain tissue of MS patient. Infiltration of proinflammatory cells through the BBB is one of major feature of MS. The BBB is a composed monolayer of endothelial cells that are mainly bound with cadherins (On et al., 2014). A hypermethylated pattern of E-cadherin (CDH_1_) may compromise BBB permeability and allow lymphocyte infiltration into brain in relapsing—remitting MS (Huynh et al., 2014; Celarain and Tomas-Roig, 2020). Inflammation is considered as primary hallmark of MS. Hypomethylation of MHC (aka. human leukocyte antigen, HLA) class I polypeptide-related sequence B (MICB) has been reported in normal appearing white matter (NAWM) (Huynh et al., 2014) and CD^4+^T cells (Graves et al., 2014) in MS patient; also, the MICB activates destruction of NK and CD^8+^T cells in MS (Meresse et al., 2006). Another study reported that demethylation of a promoter of HLA-F variant, the variant proliferates inflammatory reaction in MS (Huynh et al., 2014). Aberrant methylation pattern in MS also causes damage of myelin sheath of neurons by demyelination activated by the action of immune system (Lubetzki and Stankoff, 2014).

Studies have shown that oxidative stress and mitochondrial dysfunction contribute to the pathogenesis of MS. Based on the accumulation of lipofuscin in the neurons and glial cells found in the white and gray matter demyelination lesions, cellular senescence has been hypothesized to serve as a key player in the pathogenesis of progressive MS (Kritsilis et al., 2018). Chronic oxidative stress and inflammation accelerate age-dependent cellular senescence, and the accumulation of senescent cells above a certain threshold may contribute to senescence-dependent loss of cellular function and neurodegeneration, resulting in the secondary progression of MS (Kritsilis et al., 2018).

### Amyotrophic Lateral Sclerosis

ALS is a neurodegenerative disorder characterized by the loss or dysfunction of motor neurons in the CNS. ALS features the atrophy and progressive weakness of voluntary skeletal muscles. To date, multiple genetic mutations have been identified in non-neuronal cells, such as those in superoxide dismutase 1 (SOD1), 43-kDa TAR DNA-binding protein (TDP-43), and C9orf72, that have been linked with ALS pathogenesis. The expression of these genes is associated with neuroinflammation and immune dysregulation in ALS (Beers and Appel, 2019). A study conducted on mice in different age groups suggested that high-molecular-weight wild-type disulfide-crosslinked SOD1 aggregates during early ageing underlies motoneuron vulnerability. Surprisingly, wild-type SOD1 aggregates localize to the endoplasmic reticulum lumen, causing stress which may eventually trigger cell death (Medinas et al., 2019).

Epigenetic modifications like DNA methylation are largely unexplained in ALS. However, a cohort study of blood/CNS tissue-based analysis showed significant association of DNA methylation with age of onset and survival in genetically unexplained ALS patients (Zhang et al., 2020). Assessment of post-mortem tissue from sporadic ALS (sALS) and C9orf72 ALS (C9ALS) cases showed high level of methylation and hydroxymethylation to a cytosine (5mC and 5hmC) in the residual lower motor neurones of the spinal cord (Appleby-Mallinder et al., 2021). These data suggesting DNA methylation as a contributory factor in ALS which need further focus.

Although it is not known that mitochondrial dysfunction plays primary or secondary role in the pathogenesis of ALS, but past and recent past evidence implicated mitochondrial dysfunction in motor neuron death in ALS. Deficiency in mitochondrial transport or mitophagy may rise accumulation of abnormal mitochondria in motor neurons axon (Menzies et al., 2002; Knott et al., 2008), which can alter electron transport chain activity and impair ATP production in ALS. ALS patient’s mitochondrial analysis also revealed impairment in Ca^2+^ homeostasis, and that led to increased ROS production—related oxidative damage in motor neurons (Beal et al., 1997; Beal, 2002). Moreover, *mSOD1* genes that mimics the familial form of ALS (Gurney et al., 1994) has strong association with mitochondrial dysfunction. Overexpression of mSOD1 has been evident to activate both caspase-mediated intrinsic and extrinsic pathway in different ALS cellular or transgenic animal models (Muyderman and Chen, 2014). So far, 150 mutant forms of *mSOD1* genes have been identified. Cell lines overexpressing some forms of mSOD1 as well as transgenic animals shown activating mitochondrial proapoptotic signaling and upregulate BH3-only protein Bim and Bax (Vukosavic et al., 2000). Conversely, Bcl-2 overexpression downregulated Bim and Bax -mediated apoptotic signaling, as well as delays caspase activation in mSOD1^G93A^ transgenic mice. These data indicating mitochondrial central involvement in ALS pathology as well as proving as a valid pharmacological target. Yet it is uncertain that whether the alteration in mitochondrial biology is a primary etiology or secondary in ALS, nevertheless, mitochondrial intactness still be a determinant of motor neurons viability in ALS.

### Huntington’s Disease

HD is an age-dependent, autosomal-dominant, neurodegenerative disorder, characterized by the gradual loss of medium spiny neurons, primarily in the striatum and later in the cerebral cortex, and the accumulation of misfolded proteins in the nucleus (Cattaneo et al., 2005). A gradual increase in coding for the amino acid glutamine (CAG) and the expression of a mutant form of the huntingtin (HTT) protein (mHTT) have been reported as the key genetic features of HD pathogenesis. Normally, HTT modulates vesicular transport and synaptic transmission, whereas mHTT is neurotoxic and increased mHTT expression activates immune response-mediated neurodegeneration. Although HTT is typically abundantly expressed in neurons, during advanced stages of HD, the protein has been detected in B and T cells, monocytes, and macrophages (Li and Li, 2011).

Like the ALS, very few knowledges we have on epigenetic association with HD pathogenesis. By exploring DNA methylation-based biomarker analysis of tissue age (aka. epigenetic clock), a study showed significant association of HD and epigenetic age acceleration and significant disruption in DNA methylation levels in brain tissue (Horvath et al., 2016). Adenosine A2A receptor (A2AR) is a GPCR, highly expressed in basal ganglia and plays critical role in HD pathogenesis. Altered DNA methylation pattern has been observed in ADORA2A (genes that regulate A2AR) in HD patients (Villar-Menéndez et al., 2013). In the 5′UTR region of ADORA2A of HD patient’s putamen, the 5mC expression increased significantly while reduced 5hmC level. Interestingly, different HD mice strains (R6/1 and R6/2) striatum analysis for the same genomic region showed unchanged level of 5hmC until 12 weeks of age since when the A2AR expression reduces eventually (Villar-Menéndez et al., 2013), which is supporting the fact that changes in DNA methylation pattern is strongly associated and aggravate HD progression. The polyglutamine expansion in the HTT protein is an indicator of early stage HD. Drastic changes in DNA methylation has been found associated with this expansion and that increased mHTT expression; resulting in declined neurogenesis, cognition, and motor function in transgenic mouse and human (Ng et al., 2013; Lu et al., 2020).

Mitochondrial dysfunction, another ageing hallmark, also considered as a hallmark of HD. Mitochondrial morphology changes are depending on cell types, for example, in periphery (lymphoblast, myoblast and fibroblasts) they show an enlarged morphology, while in neurons mostly fragmented (Panov et al., 2002; Jin et al., 2013; Intihar et al., 2019). This homeostatic morphology of mitochondria alters in all cells of HD, which is manifested by decreased electron transport chain activity, oxygen consumption, Ca^2+^ buffering and decreased ATP and NAD^+^ production (Oliveira, 2010). Also, it has been suggested that mHTT-mediated mitochondrial dysfunction increases medium spiny neurons susceptibility by disrupting their energy supply (Ferrante et al., 1991). Correlating with this point, an adult rat’s striatal mitochondria showed higher sensitivity to Ca^2+^-induced membrane permeabilization than the cerebral cortex mitochondria (Brustovetsky et al., 2003), suggesting a selective metabolic stress-induced susceptibility of striatal neurons. However, cell type specific processing or localization of mHTT (Menalled et al., 2002) or brain region specific abnormal mHTT interactions (Goula et al., 2012) are also contributor of mitochondrial dysfunction and subsequent neurodegeneration in HD. Mitochondria plays a major role in the regulation of apoptosis via ROS generation and accumulation, cytochrome c release and mitochondrial permeability transition pore (mPTP) open/close homeostasis. In HD, dysregulation of two major transcription factor, p53 and PGC-1α, has frequently been reported that are related to induction and exacerbation of mitochondrial dysfunction, apoptosis and neurodegeneration (Intihar et al., 2019).

## Prion-Like Protein Concept in Ageing and Neurodegenerative Diseases

Prions (PrP^Sc^) are infectious proteins that can propagate using their β-sheet-rich conformation converting the prion protein (PrP^C^) to the aberrant form through a process of nucleated polymerization (Goedert et al., 2010). The PrP^C^ concept led in-depth understanding of neurodegenerative diseases like AD, PD, ALS, and FTD, whose common feature is protein misfolding. Until introduction of this concept, for many years it was supposed that the misfolded proteins that aggregate in diseased brain are entirely cell-autonomous event. But recent research showed that cell non-autonomous mechanisms also important for the pathogenesis of neurodegenerative diseases with intracellular filamentous inclusions. Several evidences suggested that cellular PrP^C^ plays important role in neuronal survival, outgrowth, synapse formation, maintenance and functionality, and myelinated filament formation and maintenance (Gasperini and Legname, 2014). It is supposed that ageing may alter biochemical properties and conformation of PrP^C^ and convert into pathogenic PrP^Sc^ (Gasperini and Legname, 2014). For example, loss of glycosylation can promote pathogenic PrP^Sc^ generation (Lehmann and Harris, 1997), as glycans regulate PrP^C^ folding, intracellular trafficking, and localization on neuronal surface. Also, ageing can modify subcellular localization of PrP^C^. A study showed translocation of PrP^C^ from detergent soluble fractions to lipid rafts in aged mouse (20–21 month) hippocampus (Agostini et al., 2013). These studies suggesting that age-related changes fuels PrP^C^ localization and ensue propensity to pathogenic PrP^Sc^ conversion.

Prion diseases belongs to a group of protein misfolding neurodegenerative diseases that are featured with abnormal host protein aggregation. Most of prion diseases are sporadic like Kuru and Creutzfeldt-Jakob disease, a very small percentage being familial like fatal familial insomnia. Prion diseases are very rare, conversely, AD, PD and FTD are more common and shares the protein misfolding feature. Mutations in the genes encoding APP, tau and α-synuclein cause dominantly inherited forms of AD, FTD and PD (Goedert et al., 2010). Mutations in SOD1, TDP-43 and fused in sarcoma (FUS) causes familial ALS (Rosen et al., 1993; Sreedharan et al., 2008; Kwiatkowski et al., 2009); TDP-43 mutation has also been seen in some familial cases of FTD. Huntingtin protein mutation is one major cause of HD (MacDonald et al., 1993). Although these protein misfolding diseases do not transmit between individuals as seen in the prion diseases, but the formation of tau and α-synuclein showed stereotype manner; similar to prion diseases they form in a particular regions of brain and eventually spread throughout the brain (Braak and Braak, 1991; Braak et al., 2003). Thereby, proteins exhibiting this property or specifically prion-like property has been named as “prionoids” (Aguzzi and Rajendran, 2009). Also, it has been suggested that characteristics of misfolded prion protein can be shared by other proteins central to neurodegenerative diseases (Goedert et al., 2010).

## Therapeutic Approaches

### Targeting Protein Aggregation

Neurodegenerative diseases share the common characteristic feature of protein aggregation. Neuronal protein aggregates, such as NFTs and Lewy bodies, have been detected in AD, PD, FTD, HD, and ALS, as described above (Ransohoff, 2016). Therefore, several groups have targeted protein aggregation as a therapeutic strategy (Table 1). A common strategy has been the production and clearance of Aβ by the amyloid cascade hypothesis in AD (Hardy and Higgins, 1992). According to the hypothesis, the processing of APP could have two pathways, either cleavage within AβP by the secretase that can generate peptide products that do not precipitate to form amyloid, or cleavage in the endosomal-lysosomal compartment, which can result in an intact AβP and that can precipitate to form amyloid, and can turn into NFTs and cell death, the hallmarks of AD (Hardy and Higgins, 1992). Many studies have attempted to reduce Aβ abundance by inducing immunotherapy or inhibiting γ- and β-secretases (Sevigny et al., 2016). Other have targeted tau and NFTs (Musi et al., 2018), which have been associated with several diseases, including AD, FTD progressive supranuclear palsy, and corticobasal degeneration, and their roles in other neurodegenerative diseases remain under investigation (Ransohoff, 2016; Hou et al., 2019). AD therapies targeting tau have entered phase III clinical trials, and many others are going through phase II (Cummings et al., 2018). Many researchers have suggested a mechanistic link between oxidative stress, inflammation, and neurodegeneration (Aliev et al., 2002; Blandini, 2013). Therefore, protecting against neuronal damage using phytoconstituents and dietary antioxidants might represent a potential therapeutic avenue for reducing AD risk (Essa et al., 2012). Several bioactive compounds have been suggested to modulate β-amyloid-dependent and -independent mechanisms in *in vitro* and *in vivo* AD model (Rigacci and Stefani, 2015).

**TABLE 1:** Therapeutic strategies that need further attention to intervein ageing-associated neurodegeneration.

**Target type**	**Disease and pathogen**		**Mechanism of action**	**Therapeutic purpose**
Protein aggregations	AD (Huang et al., 2020)	Aβ and tau	Aβ and tau aggregation, BACE inhibition, anti-inflammatory activity	Reduce abnormal protein accumulation, amyloid-related neuroinflammation
		Non- Aβ	Partial agonist of DD2R and 5-HT1A; microglial activation inhibitor; serotonin reuptake inhibitor	Neurotransmitter-based therapy to boost cognitive function, neuroprotection, and reduce Aβ-related neuroinflammation
	PD (Hijaz and Volpicelli-Daley, 2020)	αS	Reduce endogenously expressed αS inclusion and aggregation	Protect dopaminergic neurons and mitochondrial complex I
	ALS (Gandhi et al., 2019; Malik and Wiedau, 2020)	SOD1, FUS, TDP-43	Inhibit ubiquitylated inclusion in brain stem, spinal cord and frontal lobe of neocortex	Protect anterior horn cells and muscle fibers from motor neuron loss -mediated atrophy
	HD (Gandhi et al., 2019)	HTT	Reduce aberrant energy production and metabolic dysfunction to suppress HTT misfolding	Restore mitochondrial membrane potential and ATP balance in motor neurons
Autophagy	AD (Caccamo et al., 2017b; Xu Y. et al., 2019)		Gene therapy to induce cargo receptor SQSTM1 (p62)-mediated selective autophagy	Improve cognitive function and decrease Aβ accumulation; ameliorate microtubule associated protein tau aggregation and prion-like spreading
	PD (Moors et al., 2017)		mTOR-dependent or -independent pathway; chaperon-mediated therapeutic/s to inhibit GCase	Ameliorate αS clearance, neuronal apoptosis and moto deficits; reduce αS aggregation and synucleinopathy
	ALS (Hadano et al., 2016)		Pharmacological upregulation of SQSTM1/p62 and ALS2/alsin to inhibit ubiquitin-positive aggregations of mSOD1 in spinal neurons	Restore autophagy-autolysosomal system and protect motor neurons
	HD (Menzies et al., 2015; Fox et al., 2020)		Selective upregulation of autophagy adaptor protein Alfy to enhance autophagy-dependent clearance of proteinaceous deposits; pharmacological inhibition of calpain	Elimination of mHTT aggregation
Mitochondrial dysfunction	AD (Sorrentino et al., 2017)		Pharmacological or genetical boosting of mitochondrial proteostasis	Increase mitochondrial translation and mitophagy, and reduce amyloid aggregation
	PD (Decressac et al., 2013; Liu et al., 2017; Menzies et al., 2017)		Pharmacological upregulation of TFEB, Beclin 1 or PINK1 could potentiate mitophagy and reduce synucleinopathy	Decrease αS aggregation, αS-mediated mitochondrial dysfunction and improve cognitive function
	HD (Intihar et al., 2019)		Upregulation of PGC-1α would enhance mitochondrial biogenesis and respiration, increase electron transport chain activity and ATP synthase	Amelioration of energy deficits induced by mHTT
	ALS (Muyderman and Chen, 2014)		SQSTM1/p62 upregulation reduces mitochondrial fragmentation; PGC-1α restores mitochondrial electron transport chain in the spinal cord and reduces mSOD1	Homeostasis of mitochondrial fusion-fission and complex activity
	MS (Mahad et al., 2008)		Mitochondrial respiratory chain complex including cytochrome c oxidase	Reduce hypoxia-like tissue injury in MS

**DD2R, Dopamine D2 Receptor; 5-HT1A, 5 hydroxytryptamine 1A receptor; αS, α-synuclein; SOD1 (mSOD1), superoxide dismutase (mutant); FUS, fused in sarcoma; TDP-43, tar DNA-binding protein -43; HTT (mHTT), huntingtin (mutant); GCase, acid-β-glucosidase; TFEB, transcription factor EB; PGC-1α, proliferator-activated receptor γ coactivator 1-α.**

In PD, α-syn aggregation is widely considered to be the major pathogenic factor and is targeted to prevent the development of α-synucleinopathies. Thus, to improve PD pathology, the reduction of α-syn expression has been attempted. One study showed that small interfering (si)RNA against α-syn loaded into exosomes and injected into mouse brains and altered the levels of phosphorylated, soluble, insoluble, and aggregated α-syn (Cooper et al., 2014). That study suggested that targeting specific post-translationally modified species of α-syn species, such as phosphorylated, nitrated, oxidized, or truncated forms, may also be potential strategies for PD treatment. For example, the overexpression of kinases or phosphatases that target α-syn has been shown to be beneficial (Oueslati et al., 2013). Another plausible way to interfere with α-syn expression is by manipulating the ubiquitin-proteasome system (UPS); however, no study has yet reported on this approach in PD. Although a proteasome-activating small molecule, IU1 (1-[1-(4-fluorophenyl)-2,5-dimethylpyrrol-3-yl]-2-pyrrolidin-1-ylethanone), has been reported to reduce tau pathology, this compound has not yet been studied in an α-syn toxicity model (Lee et al., 2010). Identifying molecules able to specifically bind α-syn may help develop drugs for PD. Recently, such an approach was explored, and a compound, 3-[(3-methoxyphenyl)carbamoyl]-7-[(E)-2-phenylethenyl]-4,7-dihydropyrazolo [1,5- a]pyrimidine-5-carboxylic acid, was identified that was reported to inhibit α-syn misfolding and protect neuroblastoma cells (Xu M. et al., 2019). However, the inhibition of α-syn aggregation remains an active area of interest, with many questions that remain to be answered and barriers that must be overcome.

### Targeting Neuroinflammation

**FIGURE 4:**
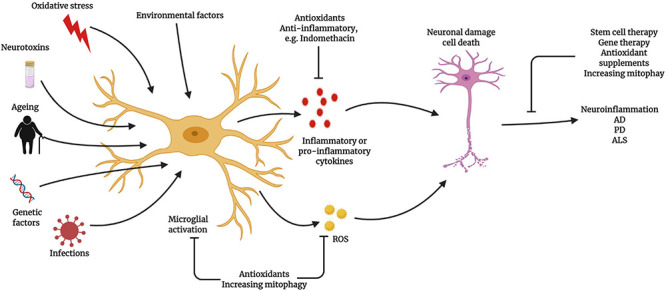
Relationship between microglial activation and neuroinflammatory diseases Microglia become activated due to ageing, exogenous or endogenous infection, oxidative stress, and genetic factors, which can lead to neuroinflammation and neurodegeneration. Activated microglia produce excessive reactive oxygen species (ROS) during ageing, triggering the activation of the nuclear factor (NF)-κB signaling cascade. Activated microglia trigger neuroinflammation to promote neuronal damage and cell death. The prevention of microglial activation and the normalization of mitochondrial function represent potential therapeutic strategies (Created with BioRender.com).

The targeting of neuroinflammatory aspects to intervene in neurodegenerative processes can have both beneficial and detrimental effects. Advanced studies examining this aspect during recent decades have demonstrated that specific neuroinflammatory genes and pathways may be associated with neurodegenerative diseases (Ransohoff, 2016). However, no therapies have emerged from this line of research, likely due to the complexity of neurodegenerative diseases, the challenges associated with designing a clinical trial, and the lack of actionable, high-throughput screening platforms (particularly for glial cells) (Ransohoff, 2016). *In vitro* glial cell cultures are widely used, but the outcomes are poorly relevant to activities and phenotypes *in vivo*. Thus, the inclusion of novel systems, such as organotypic brain-slice cultures (Masuch et al., 2016), zebrafish (Lyons and Talbot, 2014), and iPSCs (for astrocytes) (Zhang et al., 2016) might potentiate the outputs of these studies. Although non-steroidal anti-inflammatory drugs (NSAIDs) showed no effects on the risks of developing AD and PD or any cognitive benefits (Rees et al., 2011; Miguel-Álvarez et al., 2015), but interestingly, the non-aspirin NSAID ibuprofen has been associated with a 13% reduction in the risk of PD development (Rees et al., 2011), which may be useful in future. Masitinib, a tyrosine kinase inhibitor, was recently trialed (NCT02588677) on 394 ALS patients in Spain, showing promising results both alone and in synergy with riluzole (a phase 3 drug for ALS) (US National Library of Medicine, 2018). Masitinib is a novel tyrosine kinase inhibitor that targets mast cells and microglial proliferation and activation, reducing the risk of motor nerve damage (US National Library of Medicine, 2018). Observations from a decade-old neuroinflammation study suggested that therapeutics targeting glial cells might prove to be beneficial for the treatment of neurodegenerative diseases (Figure 4).

### Targeting Programmed Cell Death and Mitochondrial Dysfunction

Programmed or regulated cell death including apoptosis, necroptosis, pyroptosis, ferroptosis and others are natural process that happens in normal physiology for cell turnover and tissue homeostasis. According to Nomenclature Committee of Cell Death (NCCD), programmed cell death is a subset of regulated cell death that predestined to occur in normal physiology, for example, development (Galluzzi et al., 2015). Caspase-dependent apoptosis was the first and until now a well characterized form of programmed cell death that has visible features like shrinkage of cell, and condensation of nucleus and chromatin called pyknosis, followed by the nuclear and chromatin fragmentation called karyorrhexis. During these events several biochemical character changes sequentially, starting with the activation of pro-caspase (e.g., caspase-9) at the cytoplasm which then go through autolytic cleavage and cleave downstream effector caspases (e.g., caspase-3) (Shalini et al., 2015). Mitochondria plays a major role in this pathway and regulate caspase activation and apoptosis (Tower, 2015). Programmed cell deaths are normal physiological process that do not shift cytokine or immune balance because the apoptotic cell bodies are frequently being phagocytosed by nearby macrophages. But in ageing, this balance shift due to chronic exposure to intrinsic or extrinsic stimulus such as DNA damage, oxidative stress, hypoxia, hyperactive oncogenes and irradiation. These stimuli disrupt the mitochondrial cytoplasmic environment and up-regulate pro-apoptotic proteins, resulting in weak membrane permeabilization and excessive release of pro-apoptotic factors into cytoplasm. This including cytochrome c, Smac/DIABLO and HtrA2/Omi serine protease; while cytoplasmic cytochrome c binds and activates Apaf-1 and casepase-9 and initiates apoptotic signaling cascade.

However, ageing-related neurodegeneration like AD, PD, and HD are featured with abnormal protein aggregation that in normal physiology should’ve been cleared via macroautophagy and also including mitochondrial turnover (mitophagy). Disruption in mitophagy/autophagy could impair mitochondrial pro- and anti-apoptotic “Bcl-2 family” protein balance, resulting in programmed cell death promotion. Although precise mechanism is yet to illustrate, p53-dependent cell death has been correlated with AD (Lanni et al., 2012), while PARP-dependent pathway in PD (Lee et al., 2014). Moreover, mitochondrial protein Pink1 promotes mitophagy and ameliorate α-syn-induced neurotoxicity (Liu et al., 2017). Likewise, activation of histone acetyltransferase GCN5 by SRC-3 showed downregulation of ROS and apoptosis in PD by modulating PGC-1α translocation (Fan et al., 2020) and Bim transcription (Wu et al., 2017). However, recent study showed that inhibition of caspase-dependent apoptosis does not prevent cell death, rather shift toward necrosis (Galluzzi et al., 2015). Necrosis is an unregulated form of cell death that is characterized by cell swelling and disruption (Proskuryakov and Gabai, 2010). There is a version of necrosis, which is regulated and activated by specific stimuli like RIP1 kinsae and MLKL; the programmed necrosis is called necroptosis. Several forms of necroptosis are involved in the cleavage and nuclear translocation of mitochondrial AIF, resulting in nuclear DNA fragmentation.

Another programmed cell death mechanism is called autophagic cell death (Galluzzi et al., 2012). Macroautophagy conducts a self-eating process where cytoplasm and organelles are sequestered inside a double membrane vesicle called autophagosome, and when it fused with lysosome, the contents of autophagosome excrete into cytoplasm. The autophagic cell death is characterized by the lack of chromatin condensation and extensive cytoplasmic vacuolization. As, macroautophagy is an anti-apoptotic process, so the autophagic cell death could be beneficial as well.

Moreover, inflammasome (mostly microglial NLRP3) -mediates a novel type of programmed cell death, called pyroptosis. Very few of pyroptosis mechanism is known, but a recent study showed administration of NLRP3 inhibitor MCC950 prevented α-syn aggregation in dopaminergic neuron and mitigated motor deficits and nigrostriatal dopaminergic degeneration in PD animal (Gordon et al., 2018). Saturated fatty acids from diet could also trigger pyroptosis by increasing TLR4 and cleavage of caspase-1 from caspase-11, resulting in enteric neuropathy (Ye et al., 2020). Involvement of the multiprotein complex, inflammasome, has also been evident in AD pathology (Feng et al., 2020).

Dixon et al. (2012), another form of programmed cell death mechanism has been proposed where cell death occurs by the small molecule erastin that inhibit influx of cystine and leads to glutathione depletion and inactivation of phospholipid peroxidase glutathione peroxidase 4 (GPX4) (Yang et al., 2014). This form has been named as ferroptosis, as it is characterized by the iron-dependent accumulation of lipid hydroperoxides to lethal level. Ferroptosis is robustly dysregulated by PKCα activation, so a study proposed that iron chelator, Fer-1, derivatives or PKC inhibitor might be a good candidate for PD therapy (Do Van et al., 2016; Guiney et al., 2017).

Cell death program is an old but emerging target for neurotherapeutics. But other than apoptosis, which has been characterized at molecular level so far, the extent of the other forms of regulated cell death program under normal physiology are unknown. There are several susceptibilities that could cause mis-regulation of these cell death program during ageing. Including, disruption in immune system, disrupted balance of multiple pro- and anti-apoptotic factors, maladaptive signal transduction, and finally and most importantly mitochondrial dysfunction (Tower, 2015). Several aspects made mitochondria as the most important regulator of cell death program mis-regulation and also made an emerging target for therapeutic development, for example, ageing decreases cellular turnover of highly mitotic tissues (Kavathia et al., 2009) that reduces mitophagy rate, resulting in loss of membrane permeabilization and increased cell death. A brief list of pharmacological therapeutics is given in Table 2 that are potential candidates in age-related neurodegenerative disease treatment.

**TABLE 2:** List of current and prospective inhibitors of different programmed cell death.

**Cell death program**	**Agent**	**Target**	**Disease**
Necroptosis	Necrostatin-1	RIPK1	Ischemic brain injury (Degterev et al., 2005), AD (Caccamo et al., 2017a)
	Ponatinib and pazopanib	RIPK1, RIPK3 and TAK1; inhibit TNF-α-induced phosphorylation of MLKL	Ischemia-reperfusion injury (Fauster et al., 2015)
	HS-1371	Specifically inhibits TNF-induced necroptosis, not apoptosis, by selectively suppressing RIPK3	Disease involving RIPK3 hyperactivation (Park et al., 2018)
	Necrosulfonamide	Specifically blocks MLKL downstream RIPK3	MLKL and RIPK3 hyperactivation (Sun et al., 2012)
Pyroptosis	Minocycline	Downregulate NLRP3 and TLR2 activity in microglia and attenuate Aβ deposition; however, 2 years clinical trial on AD showed no benefit on cognitive function	AD (Garcez et al., 2019; Howard et al., 2020)
	Edaravone	Scavenge ROS and alleviate neuronal damage in ischemic stroke; increase SOD-2 and reduce NLRP3 in AD microglia	Ischemic stroke (Yoshida et al., 2006); AD (Wang H. M. et al., 2017)
	Baicalin	TLR4/NF-κB pathway and NLRP3 activation	AD (Jin et al., 2019) and PD (Lin, 2011)
Ferroptosis	D-PUFA, ferrostatin, liproxstatin, CoQ10	Blocks lipid peroxidation	Ferroptosis-mediated neurodegeneration (Stockwell et al., 2017)
	Dopamine	Inhibits GPX4 degradation	Stockwell et al., 2017
Apoptosis	β-carotene	Balance Bax/Bcl-2 and reduces cytochrome c release	Cerebral ischemia, AD and PD (Park et al., 2020)

**RIPK, receptor-interacting serine/threonine-protein kinase; TAK-1, transforming growth factor-β-activated kinase 1; TNF-α, tumor necrosis factor- α; MLKL, mixed lineage kinase domain-like protein; NLRP3, NLR family pyrin domain containing 3; TLR4, toll-like receptor 4; NF-κB, nuclear factor κ-light-chain-enhancer of activated B cells; GPX4, glutathione peroxidase 4; Bax, Bcl-2-associated X protein; Bcl-2, B-cell lymphoma 2.**

### Dietary Supplements as a Therapeutic Target

NAD^+^ is a key cofactor involved in cellular energy metabolism and is involved in several other biological activities, including the maintenance of mitochondrial health, energy homeostasis, stem cell renewal, DNA repair, and neurological function (Fang et al., 2017). Decreased NAD^+^ has been reported in both mice and humans with advanced age. An *in vivo* NAD^+^ assay demonstrated an age-dependent decline in NAD^+^ activity in the human brain (Zhu et al., 2015). NAD^+^ replenishment may delay the onset of premature ageing and ageing-associated neurodegeneration (Fang et al., 2017).

Additionally, increasing evidence has suggested that certain dietary patterns support brain health, such as the increased intake of fruits, vegetables, and fish (Kang et al., 2005; Barberger-Gateau et al., 2007). One meta-analysis examining the intake of the Mediterranean diet, which includes large quantities of fruits, vegetables, whole grains, fish, and unsaturated fatty acids, reported a reduced probability of developing cognitive impairment (Wu and Sun, 2017). The inclusion of olive oil and nuts in the diet improved cognitive function (Valls-Pedret et al., 2015), as reported by another group. Moreover, different cross-sectional studies showed that the Mediterranean diet also has the potential to reduce the risk of developing depression among older adults (Moore et al., 2018). The relationships between polyphenols, brain health, and ageing represent an emerging area of study. Several studies have shown that the regular intake of specific or random polyphenols is associated with improved cognitive function (Schaffer et al., 2012; Brickman et al., 2014; Azam et al., 2019). For example, the supplementation of cocoa flavanol for up to 2 months improved the cognitive performance of an older adult group (Mastroiacovo et al., 2015). Long-chain fatty acids, including *n-3* polyunsaturated fatty acids (PUFAs), eicosapentaenoic acid (EPA), and docosahexaenoic acid (DHA), have been shown to be beneficial for cognitive and mental health in some studies (Grosso et al., 2014). One meta-analysis examining *n-3* fatty acids concluded that these fatty acids were protective for a specific subset of cognitively impaired patients, although this study did not identify any significant relationship between fatty acids and AD (Mazereeuw et al., 2012). The increased dietary intake of protein has also been positively associated with improved non-verbal and verbal learning and memory and a reduction in the risk of mild cognitive impairment (MCI) or dementia (Roberts et al., 2012). The important role played by nutrition in a healthy life is generally accepted and has been well studied. Future studies that focus on this emerging field should attempt to identify thresholds for optimizing the nutritional requirements that may contribute to slowing or preventing the development of cognitive decline or other ageing-associated complications.

### Other Targets

Various cellular and molecular processes that promote or delay ageing could be targeted by pharmacological interventions to treat numerous neurological diseases. Study areas could include oxidative stress, calorie restriction, telomerase protection, autophagy, stem cell renewal, and epigenetic modifications (see Figures 1, 4). Calorie restriction increases mitophagy and cellular proliferation via a pathway that involves mTOR signaling. Many antioxidants, such as flavonoids, carotenoids, quercetin, curcumin, and resveratrol, have been shown to reduce the risks of AD, PD or ALS development (Vaiserman et al., 2016; Azam et al., 2019). A recent study showed that genomic instability and DNA damage activates cyclic GMP-AMP synthase (cGAS), a DNA sensor that triggers innate immune responses, linking DNA damage with inflammation, cellular senescence, and cancer (Li and Chen, 2018); however, the link between AD and the activation of cGAS has not been investigated yet.

## Conclusion

Currently, various treatment strategies are being investigated to slow or reverse ageing-associated diseases; unfortunately, no preventive or effective treatments have yet been identified. The major challenge associated with this process, thus far, remains the lack of highly efficient disease models of neurodegeneration. In this review, various ageing hallmarks were discussed, most of which have been associated with neurodegenerative diseases. We propose that future studies of neurodegenerative diseases should focus on these hallmarks of ageing and that ageing models should be developed that show neurodegenerative disease phenotypes. Although this paper primarily focused on DNA damage, cellular senescence, and mitochondrial dysfunction, studies examining the relationships between the nucleus and mitochondria would reveal the mechanistic links between ageing and neurodegeneration. Other hallmarks, such as proteostasis, epigenetic deregulation, and telomerase inactivation, are also important. The loss of proteostasis results in proteasomal and autophagy defects in both AD and PD, resulting in inflammation and senescence (López-Otín et al., 2013). Metabolic dysfunction has been shown to be associated with mitochondrial dysfunction, oxidative stress, and NAD^+^ levels (Babbar and Sheikh, 2013).

Although cross-talk between inflammatory pathways and neurodegeneration has been recognized for the past two decades, very few therapeutic strategies have emerged from this line of research due to the lack of a high-throughput screening platform. To harness the therapeutic potential of inflammatory pathways, a better understanding of the neuroprotective role played by TNF-α and NF-κB remains necessary, and new models must be developed that are able to recapitulate microglia-induced neurodegenerative phenomena *in vivo* (Klünemann et al., 2005; Ransohoff, 2016). However, neurodegenerative diseases are complex to decipher, and their central mechanisms are further complicated by the interactions that occur between genetic and environmental factors, which drive disease progression. A single-pathway-oriented therapeutic intervention might not be sufficient for the treatment of these complex disease, although combination therapies may be successful. Identifying functional links between neurodegenerative diseases and ageing hallmarks could reveal new therapeutic avenues. A multi-target, evidence-based approach associated with non-pharmacological approaches, such as lifestyle modifications, may slow neurological disease progression in older individuals.

## Author Contributions

SA and D-KC conceptualized and designed the study plan. SA and MH reviewed the literature, collected the data, and wrote the manuscript. RB and I-SK revised the final draft of the manuscript and made necessary corrections. All authors contributed to the article and approved the submitted version.

## Conflict of Interest

The authors declare that the research was conducted in the absence of any commercial or financial relationships that could be construed as a potential conflict of interest.

## Publisher’s Note

All claims expressed in this article are solely those of the authors and do not necessarily represent those of their affiliated organizations, or those of the publisher, the editors and the reviewers. Any product that may be evaluated in this article, or claim that may be made by its manufacturer, is not guaranteed or endorsed by the publisher.

## Abbreviations

AD: Alzheimer’s Disease
PD: Parkinson’s Disease
ALS: amyotrophic lateral sclerosis
FTD: frontotemporal lobar dementia
A β: amyloid-beta
NFT: neurofibrillary tangle
LBD: Lewy body dementia
NER: nucleotide excision repair
BER: base excision repair
PINK1: PTEN-induced putative kinase protein 1
BNIP3L/NIX: BCL2/adenovirus E1B 19 kDa protein-interacting protein 3-like
NIX: Nip3-like protein X
*Gpld1*: glycosylphosphatidylinositol (GPI)-specific phospholipase D1
DAM: disease-associated microglia
ApoE: apolipoprotein E
GWAS: genome-wide association study
NGS: next-generation sequencing
NSPCs: neural stem/progenitor cells
SA- β-gal: senescence-associated β-galactosidase
FPR2: formylpeptide receptor 2
MAPK: p38 mitogen-activated protein kinase
α-synuclein: α-syn
*Drp1*: dynamic-related protein 1
iPSC: inducible pluripotent stem cell
NLRP3, NOD-: LRR- and pyrin domain-containing protein 3
SOD1: superoxide dismutase 1
NAD^+^: nicotinamide adenine dinucleotide
MCI: mild cognitive impairment
SNPs: single- nucleotide polymorphisms
TNF- α: tumor necrosis factor- α
NF- κ B: nuclear factor κ B.
